# Repeat positive SARS-CoV-2 RNA testing in nursing home residents during the initial 9 months of the COVID-19 pandemic: an observational retrospective analysis

**DOI:** 10.1016/j.lana.2021.100054

**Published:** 2021-08-21

**Authors:** Jillian N. Armstrong, Lauren Campbell, Terry Rabatsky-her, Vivian Leung, Sunil Parikh

**Affiliations:** a Department of Epidemiology of Microbial Diseases, Yale School of Public Health, New Haven, CT, USA; b Connecticut Department of Public Health, Hartford, CT, USA

**Keywords:** SARS-CoV-2, COVID-19, diagnosis, RT-PCR, reinfection, Nursing Homes, repeat positive tests, long-term care facility, skilled nursing facility, Immunity

## Abstract

**Background:**

Nursing homes are high-risk COVID-19 settings with residents who are typically older and have multiple comorbidities. SARS-CoV-2 testing occurs frequently in nursing homes, with public health guidance suggesting that repeat testing is generally not warranted in the 90 days following initial positive test results. Interpretation of repeat positive tests beyond 90 days is challenging and the consequences of decisions following these tests are significant.

**Methods:**

We utilized a surveillance system for COVID-19 to identify Connecticut nursing home residents who tested positive for SARS-CoV-2 by RNA-based testing ≥ 90 days after initial positive results. We analyzed statewide nursing home testing data over a 9-month period, from the first Connecticut nursing home case identified on March 15 through December 15, 2020, when nursing home COVID-19 vaccinations began in Connecticut.

**Findings:**

We identified 156 residents (median age 75 years) with positive RNA-based PCR tests occurring ≥90 days after an initial positive test. Residents with repeat positives tests represented approximately 2.6% (156/6,079) of nursing home residents surviving beyond 90 days of their initial SARS-CoV-2 diagnosis statewide since the start of the pandemic, with a median time to repeat positivity of 135 days (range 90–245 days). Deaths were reported in 12.8% (20/156) of residents following the repeat positive test, with 80% (16/20) having one or more intervening negative RT-PCR tests prior to the repeat positive test.

**Interpretation:**

Our analysis suggests that repeat positive testing in nursing home populations may exceed those reported in younger age groups. Repeat positive tests beyond 90 days may accompany severe outcomes, and should be prospectively investigated with genomic, virologic and additional data, when feasible. Data shed light on the duration of protective immunity following natural infection in this subset of largely elderly and medically frail individuals.

**Funding:**

This work was conducted in the context of the Connecticut DPH COVID-19 response and not supported by specific funding.

**Research in Context**

**Evidence before this study**

Confirmed cases of SARS-CoV-2 reinfection remain relatively rare, although repeat positive SARS-CoV-2 tests have been increasingly reported in the literature and in clinical practice. Population based studies have reported rates of repeat positive tests generally below 1%, with a report from Denmark suggesting a rate of 0.65%, and higher rates in those over 65 years of age. Residents of nursing homes represent the highest risk group for COVID-19 morbidity and mortality. A primary means of infection prevention and control in these settings is frequent SARS-CoV-2 testing, and CDC guidance recommends against repeat testing within 90 days of an initial positive test unless significant clinical suspicion for reinfection exists. If tests are positive, additional investigation is recommended, but is often challenging in the setting of a public health response of this size and scope. Data on the rates of repeat test positivity in nursing home settings is limited to a small number of outbreaks but suggest that the frequency may be higher than in the general population.

**Added value of this study**

We utilized a statewide surveillance system to identify SARS-CoV-2 test results of residents in Connecticut's 212 nursing homes for the initial 9 months of the pandemic, prior to the onset of vaccination. Approximately 11,644 SARS-CoV-2 unique cases were recorded, and of those that survived beyond 90 days of their initial diagnosis, 2.6% were found to have repeat positive tests at a median of 4.5 months. Most notably, 12.6% of residents with repeat positive tests died shortly thereafter (median 8 days after repeat positive test), and 80% had one or more intervening negative tests. While genetic sequencing data was not obtained, available data suggest that these cases may have represented SARS-CoV-2 reinfections with associated poor outcomes in this elderly and largely frail population.

**Implications of all the available evidence**

Taken together, our data support that repeat testing should continue in individuals living in nursing homes once 90 days have elapsed since their initial positive SARS-CoV-2 test. Repeat positive tests are frequent in this demographic and may indicate true reinfection and risk for poor outcomes. Moreover, the high frequency of repeat positive tests in this group, as compared to younger populations or community dwelling elderly, suggest that immunity may wane more quickly following natural infection in this demographic. These data have significant implications for assessing the continued risk of SARS-CoV-2 in residents of long-term care settings following natural infection.

Alt-text: Unlabelled box

## 1 Introduction

In the United States, skilled nursing facilities (SNFs) have been an epicenter of the coronavirus-19 disease (COVID-19) pandemic, accounting for approximately one out of three deaths nationwide. Early in the pandemic, the Northeast United States was particularly hard hit, and by mid-December 2020, just prior to the start of COVID vaccine deployment in Connecticut, nursing homes in the state had over 11,644 cases, with over 75% (8,777/11,644) occurring before mid-July [1].

When increased availability of severe acute respiratory syndrome coronavirus 2 (SARS-CoV-2) RT-PCR testing occurred in April 2020, Connecticut SNFs implemented widespread frequent point prevalence surveys (PPS) of residents and staff, particularly in the setting of outbreak containment [2]. In the context of frequent testing, a large number of repeat positive tests have occurred, the results of which have posed challenges for nursing homes and public health officials to interpret in the context of a pandemic response.

Repeat positive SARS-CoV-2 RT-PCR tests may represent true reinfections or persistent shedding of viral RNA in the absence of reinfection. Several occurrences of confirmed reinfection by genomic sequencing have been reported since mid-2020, though their frequency appears to be rare. Reports from the European CDC, Qatar, and the UK suggest that reinfection is rare, and that no onward transmission from confirmed repeat positive cases had yet been documented in the scientific literature [3], [4], [5]. In October 2020, the U.S. Centers for Disease Control and Prevention (CDC) reported that reinfection is uncommon during the initial 90 days following initial infection onset, and recommended that testing not be repeated during this time if an individual is asymptomatic [4]. This public health recommendation was supported recently in a prospective study [6]. If reinfection is suspected after this time (or before 90 days in the presence of symptoms), recommendations are to obtain samples for genetic testing, and incorporate information such as cycle threshold (Ct) values and clinical status to help determine the likelihood of reinfection [7].

Such determinations in SNFs have been challenging, and nearly always must be made without real-time genomic data. The consequences of decisions are significant, as responses may range from individual quarantine to facility-wide PPSs. Equally importantly, the timing and outcomes following repeat positivity may shed light on the duration of immunity following natural infection, which may differ in SNFs due to immunosenescence in the elderly and otherwise compromised immune systems in the setting of multiple comorbidities. To help provide insight into the frequency of repeat positives in SNFs, we investigated the frequency of repeat positive SARS-CoV-2 tests in Connecticut nursing home residents between March 15, 2020, the 1^st^ nursing home case in Connecticut, and December 15, 2020, just prior to the start of vaccinations in Connecticut nursing homes. We report on the results of SARS-CoV-2 tests performed during the time between positive tests, as well as demographic and clinical data, where available.

## 2 Methods

### 2.1 SARS-CoV-2 surveillance system in Connecticut

The total number of SARS-CoV-2-positive cases in nursing homes was derived from the publicly available data from the Connecticut Department of Public Health (CT DPH) and the National Healthcare Safety Network (NHSN) [1]. The Connecticut Electronic Disease Surveillance System (CTEDSS) maintains records of confirmed SARS-CoV-2 RT-PCR tests statewide since the onset of the pandemic in March 2020 (https://edss.dph.ct.gov). SARS-CoV-2 RT-PCR and antigen test positivity was determined by each individual laboratory following product guidance specific for each test and platform. From CTEDSS, data was extracted for all tests from individuals living in a congregate facility, including a secondary data review process, and further refined to identify those living within nursing homes. For additional individual-level SARS-CoV-2 testing and demographic details, a web-based portal for nursing home COVID-19 case reporting maintained by CT DPH since mid-April 2020 was utilized [1]. Symptomatic status was determined by nursing home staff or hospital data, when available [8]. Additional data were obtained by study team members via phone calls to both nursing homes and laboratory personnel.

Data collected for this research activity qualified as exempt from review by the CT DPH Human Investigations Committee (HIC) under federal guidelines 45 CRF § 46.102(l) (2). Activities including data extraction from surveillance databases, additional data collection on clinical characteristics and RT-PCR Ct values were deemed not to be research by CT DPH and Yale Human Investigations Committees.

### 2.2 Identification of deaths in nursing home residents

Death information obtained by the Office of Chief Medical Examiner (OCME) was recorded in CTEDSS, and extracted on March 22, 2021 to identify all individuals who died prior to December 31, 2020 that had repeat positive tests ≥90 days following an initial positive test. Data on cause of death were obtained from hospitals or nursing homes.

### 2.3 RT-PCR testing information

Case dates correspond to the date of specimen collection. Specimens were run at several labs and on multiple platforms. Specimen sampling sites and Ct were obtained, when available. In the case of repeat positive tests which were initially obtained via antigen-based tests, confirmatory RT-PCR results were obtained and reported, when available [9].

### Role of funding source

This work was not supported by specific funding, as it was carried out in the context of the CT DPH COVID-19 public health response.

## 3 Results

An accurate total nursing home census at the onset of the pandemic was not available, though the range in any given week from June 23 to December 15, 2020 was 17,551 to 18,417 residents. The total number of SARS-CoV-2-positive cases in Connecticut's approximately 212 nursing homes was reported as 11,644 cases and 3,315 deaths for the 9-month period between March 15 and December 15, 2020 [1].

Repeat positive SARS-CoV-2 tests occurring ≥90 days following an initial positive test were found in n=181 residents during the study period. Of those with repeat positive tests, n=25 were excluded due to subsequent negative confirmatory PCR testing within three days of their positive antigen test. Data on symptom status were available for 17/25 of these residents, 16 of whom were listed as having been asymptomatic at the time of testing. After exclusion of the 25 residents with repeat positive findings on antigen testing but negative PCR confirmation, there were n=156 residents with repeat positive RT-PCR results.

During the final 90 days of the study period (September 15 to December 15, 2020), 2,797 cases were recorded in nursing homes. During the initial 90 days of the study period (March 15 to June 15, 2020), 2,768 nursing home deaths were recorded in CTEDSS. As neither of these groups could have been in the study period for ≥90 days, a maximum of 6,079 case-patients survived beyond June 15, 2020, and could retest positive for SARS-CoV-2 ≥90 days prior to the study end [1]. Therefore, our most conservative estimate for the rate of repeat positive tests among nursing home resident cases is 2.60% (156 cases with repeat positive tests among 6,079 case patients) during the study period. A total of 1920 tests were conducted among these individuals over the study period (median 12 per resident). Residents with repeat positive tests were of a median age of 75 years (range 36–105). Table 1 describes demographics and other characteristics of this cohort.

**Table 1:** Demographics and characteristics of Connecticut nursing home residents with a repeat positive SARS-CoV-2 test ≥90 days after an initial positive test (March 15 - December 15, 2020)

**Total # residents with repeat positive test**	156
**Total # nursing homes with repeat positive residents**	81
**Median resident age in years (IQR)**	75 (66,86)
**Resident sex (No. (%) female)**	91 (58)
**Resident racial category (No. (%))**	
White	112 (72)
Black	24 (15)
Hispanic	13 (8)
Other	7 (5)
SARS-CoV-2 tests conducted in residents with repeat positive tests*	1920
Total # RT-PCR tests (%)	1861 (97)
Median total # tests per individual (IQR)	12 (8,16)
Median total # tests per individual after 90 days elapsed (range)	9 (1,25)
**Sampling location of repeat positive test**	
Nasopharyngeal swab	145 (93)
Oropharyngeal swab	2 (1)
Nasal swab	6 (4)
Unspecified	3 (2)
**Median duration between initial positive test and 1^st^ repeat positive test after 90 days (IQR)**	135 (110,185)
**Reported reason for testing at time of repeat positive test (No. (%))**⁎⁎	128 (82)
Routine surveillance	64 (41)
Hospital Admission (any reason)	35 (22)
Symptomatic (concern for COVID-19)	15 (10)
Testing due to outbreak/contact investigation at facility	7 (4)
Appointment/Discharge from facility or hospital	7 (4)
Unknown	28 (18)
**Number with symptoms at time of initial positive test (%)**⁎⁎	98/147 (67%)
**Number with symptoms at time of repeat positive test (%)**⁎⁎	44/124 (35%)

Abbreviation: IQR, interquartile range

⁎ SARS-CoV-2 tests were run at n=51 different labs, and on n=14 different RT-PCR platforms.

⁎⁎ The total number is lower than the group total (n=156) due to availability of clinical data.

Supplemental Figure 1 displays the test results for all tests available in CTEDSS for individuals with repeat positive tests ≥90 days after the initial tests beginning from their initial positive test through December 18, 2020. Initial positive tests were all RT-PCR-based. The median duration between the initial and 1^st^ positive test ≥90 days was 135 days, with a range of 90–245 days. Ct values from 38 laboratories were retrievable for 71/156 initial tests and 51/156 repeat positive tests. Of the repeat positive tests, 27.5% (14/51) had Ct values <33, a threshold recommended by CDC for further investigation of suspected reinfection [7]. Data on the symptomatic status at first and repeat positive test, as determined by nursing home staff, as well as the reasons for testing at the time of the repeat test are in Table 1.

**Figure 1:**
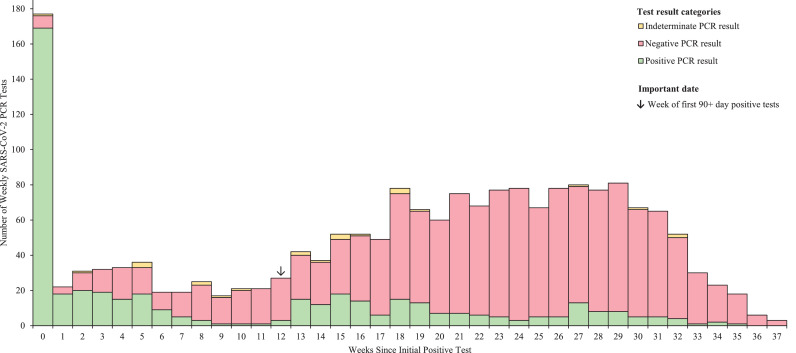
Pattern of RT-PCR results in n=156 residents testing positive ≥90 days following the initial positive SARS-CoV-2 RT-PCR test. Histogram of the number of PCR tests conducted in the weeks following an initial positive SARS-CoV-2 RT-PCR for n=156 nursing home residents who tested positive ≥90 days following an initial positive SARS-CoV-2 RT-PCR test. Yellow, red, and green represent indeterminate, negative, and positive PCR test results respectively. Individuals may have had one or more tests during a week. The arrow indicates the week of the first 90+ day positive SARS-CoV-2 tests.

Review of death certificate data in mid-March, 2021 for all n=156 individuals revealed that n=20 of 156 repeat positive cases died as of December 31^st^, 2020 (Table 2 and Figure 1). COVID-19 was noted on death certificates as either a primary or secondary cause of death, as determined by the nursing home or healthcare facility. Days between the first positive test and initial repeat positive in individuals who died ranged from 93 to 217 days (median 169 days). The median number of days between repeat positive test and death was 8 days (interquartile range 2 to 15 days). The proportion of individuals who had one or more intervening negative tests between the initial and repeat positive was 16/20 (80%) in those who died (Figure 1), 110/136 (81%) in those who did not die, and 126/156 (81%) in the overall study population. The median duration between initial positive and most recent intervening negative tests in individuals who died was 87 days (interquartile range 30,126). Ct values for repeat positive tests were available for six of 20 residents who died, with 50% (3/6) having a Ct <33. Only five of the 20 individuals who died (IDs O, F, T, B, and D in Table 2, Figure 1 and Supplemental Figure 2) had a subsequent negative test after their repeat positive; with death occurring 0, 4, 38, 79, and 118 days after the initial repeat positive test, respectively. Detailed information on the clinical status and hospitalizations for residents who died was incomplete and therefore not reported.

**Table 2:** Demographics and characteristics of nursing home residents who died following a repeat positive SARS-CoV-2 test result ≥ 90 days after an initial positive test (March 15 - December 31, 2020)

ID	Age	Sex	Race	Date initial positive	Days between initial and repeat infection	Negative test in between	Ct value of repeat positive test *	Days from most recent positive test to death	Symptoms at time of repeat positive test ⁎⁎
**A**	73	M	NHW	4/18/2020	93	No	N: 36•6	0	Unknown
**B**	72	M	NHW	4/17/2020	98	Yes		79	Yes
**C**	89	M	NHW	5/1/2020	101	No		1	Yes
**D**	98	F	NHW	5/20/2020	104	Yes	N2: 40•0; E: 42•3	118	Yes
**E**	91	F	NHW	5/24/2020	105	No		2	Yes
**F**	71	M	NHW	5/20/2020	105	Yes	S: 23•9; N: 24•9; ORF1ab: 23•3	4	Yes
**G**	74	M	NHMR	4/10/2020	109	Yes		2	Unknown
**H**	69	F	NHW	4/27/2020	122	No		4	Yes
**I**	99	F	H	5/8/2020	131	Yes	N: 36•0; ORF1ab: 36•3	13	Yes
**J**	92	F	NHW	4/24/2020	153	Yes		1	No
**K**	90	F	NHW	4/27/2020	184	Yes	S: 13•4; N: 13.4; ORF1ab: 12•5	6	Yes
**L**	82	F	NHW	5/13/2020	189	Yes		7	Yes
**M**	91	M	NHW	4/27/2020	190	Yes		11	Yes
**N**	91	F	NHW	4/27/2020	190	Yes		15	Yes
**O**	66	F	NHW	4/3/2020	196	Yes		0	Unknown
**P**	87	F	NHW	4/22/2020	203	Yes	S: 14•8; N: 16•5; ORF1ab: 15•2	10	Yes
**Q**	84	F	NHB	5/12/2020	211	Yes		7	Unknown
**R**	77	F	NHW	5/12/2020	211	Yes		14	Yes
**S**	94	F	NHW	5/12/2020	217	Yes		3	Yes
**T**	83	F	NHW	4/5/2020	217	Yes		38	Yes

Abbreviations: Ct, cycle threshold; F, female; M, male; H, Hispanic; NHB, Non-Hispanic Black; NHMR, Non-Hispanic mixed-race; NHW, Non-Hispanic White

⁎ RT-PCR kits for SARS-CoV-2 often include targets for one or more structural genes, such as the envelope (E), spike (S) protein, and nucleocapsid (N, N2) genes, or species-specific targets such as the open-reading frame (ORF1ab) genes. Interpretation of Ct value results is kit-specific.

⁎⁎ Data on clinical status is incomplete and based on reports either in CTEDSS or obtained via phone from nursing home or healthcare facility.

## 4 Discussion

We present a comprehensive dataset of 9 months of statewide RT-PCR data from residents living in Connecticut's 212 nursing homes from the start of the COVID-19 pandemic through the end of 2020. Our data precedes the start of vaccination, providing an assessment of viral testing patterns following naturally acquired infection in this important and highly vulnerable population. Our study was framed in the context of “90-day” guidance from the CDC for reinfection assessment [10]. Repeat positive tests beyond 90 days, while infrequent, occurred in approximately 2.6% (156/6,079) of nursing home residents with RT-PCR-confirmed SARS-CoV-2 at a median of ∼5 months post-initial infection, and repeat positive tests could be seen out to eight months.

The frequency of repeat positives in the nursing home setting was higher than that reported in other studies of repeat testing in younger populations or community dwelling elderly adults, where repeat positivity rates have generally been below 1% [[3], [4], [5], [6],[11], [12], [13]]. A population-based study in Denmark found 0.65% of the population tested positive in the 1^st^ and 2^nd^ surges [11]. While the frequency of repeat positives was not explicitly reported in elderly in this study, it was noted to be higher in those >65 years old, with 47.1% protection against infection in the elderly as compared to 80.5% in the general population. Studies with a similar demographic and setting are thus far limited. One study of subsequent outbreaks in two nursing homes in the United Kingdom found that 1.1% of residents were found to have repeat positive PCR tests over the two outbreaks [14]. In the United States, a study of two outbreaks in a Kentucky nursing home separated by three months found that five residents had probable reinfections, and that severity was worse in all residents, with one death reported [15]. Our data, taken in context of emerging literature, support that elderly and often medically frail adults, particularly in congregate settings, could be at higher risk of reinfection with SARS-CoV-2.

**Figure 2:**
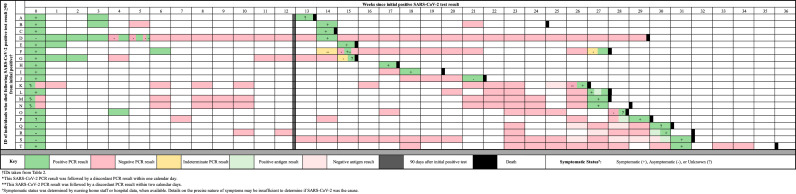
Detailed testing results for those who died before December 31, 2020 following repeat positive SARS-CoV-2 RT-PCR testing. Weekly SARS-CoV-2 testing results for nursing home residents who died before December 31, 2020 following a repeat positive SARS-CoV-2 RT-PCR test ≥90 days following an initial positive test. Green, red, and yellow represent positive, negative, and intermediate PCR test results respectively. Black hashmarks represent antigen test results. Gray represents the 90-day threshold after the initial positive SARS-CoV-2 RT-PCR test. Black represents the date of reported death. Weeks with discordant results are represented by multiple colors according to the number, results, and sequence of the discordant tests

Following from this report in Kentucky, our findings are striking in that that 12.6% (20/156) of repeat positive cases in Connecticut nursing homes were temporally associated with death at a median of 8 days following repeat positive testing. Deaths in these individuals occurred at a median of 5.6 months after initial infection, but were seen as early as three months following first infection [15]. While we lack conclusive evidence that these twenty cases represent true reinfections, several lines of supporting evidence suggest that true reinfection may have occurred in some of these cases: 1) 75% (15/20) of cases died within 2 weeks of a repeat positive test; 2) 80% (16/20) had intervening negative RT-PCR tests following their initial SARS-CoV-2 diagnosis in the 1^st^ surge, and frequently had multiple negative tests extending for months prior to repeat positive testing (Figure 2); 3) Ct values, in the limited cases where they were available, were <33 in 27.5% (14/51); and, 4) all individuals were reported as COVID-related deaths by the nursing home or healthcare facility.

Due to the frequency of testing, our data also demonstrate the complexity and stochasticity of testing results in this population (Supplemental Figure 2). We identified n=25 residents whom positive antigen tests were followed by a negative confirmatory PCR test within a few days, providing support for CDC antigen test recommendations in nursing home settings [9]. Ct values in intermittently positive individuals were generally high (above 33; data not shown), and were quickly followed by negative tests [7]. Definitive diagnosis of reinfection requires sequencing, and can additionally be aided by sampling for viral culture [3,7]. As noted recently, such data is difficult to obtain due to the public health burden and lack of testing and sequencing capacity throughout the pandemic, particularly in the 1^st^ surge [16].

An additional explanation for these stochastic results is persistent or intermittent shedding of viral RNA, which may be due to a less effective immune response, viral sequestration or latent reactivation. Although rarely culturable beyond 10 days, the median duration of RNA detection following infection is reported as 18 days, with a duration of up 185 days in the upper respiratory tract [4,10,[17], [18], [19], [20], [21], [22]]. While reassuring, numerous reports suggest that an independent risk factor for persistent positivity is older age, especially age >60-65 years [17,23]. Intriguingly, immunologic studies suggest that antigen persistence is driving continued memory B cell maturation following infection, and that the gut may be a source of continued nucleic acid [24]. Further work to elucidate whether the gut or other tissues can sequester SARS-CoV-2 over extended periods is warranted.

Taken together, data suggest that the durability and potency of the acquired immune response to natural infection in this largely elderly and frail population may be less robust than in younger or similarly aged community dwelling adults. Although continued maturation of memory B cell responses and immunity following natural infection appears to last up to six months or longer [5,[24], [25], [26], [27], [28]], antibody titers have been found to decay by 4 to 6 months post-infection [24], with CD4^+^ and CD8^+^ T cell-specific responses declining with a half-life of 3-5 months [25,28,29]. While detailed studies looking at kinetics and effectiveness of memory responses to SARS-CoV-2 in the elderly are not yet available, studies of immunosenescence suggest that responses may be less durable and robust at the extremes of age and in the setting of multiple comorbidities [30].

Our study is subject to several important limitations. As noted above, limited Ct values, lack of genetic sequencing and culture data, and incomplete clinical data make it difficult to confirm whether repeat positive RT-PCR tests represent true reinfections. In addition, our data are likely an underrepresentation of the overall frequency of repeat positive tests. Firstly, we selectively sought testing results only from residents testing positive ≥90 days beyond their initial test. Figure 1 suggests that results prior to 90 days also show stochasticity, and thus we are unable to comment on the frequency of repeat positives before 90 days in SNF residents. Secondly, while we removed 2,768 residents that died before June 15, an additional approximately 547 deaths occurred between June 15 and December 15, 2020, and were not removed from the denominator. Inclusion of these individuals results in a frequency of repeat positives in our population of 2•82% (156/5,532). Thirdly, the age, sex, and racial demographics of the population included in this investigation might affect generalizability to other SNF populations or to elderly living in community settings.

As the pandemic continues, an accurate interpretation of repeat positive tests will be critical, as they can result in re-institution of individual isolation and facility-wide testing. Most importantly, our data suggest that following initial infection, this particularly vulnerable demographic may be at higher risk for repeat infection with SARS-CoV-2 and possible severe outcomes at that time. This risk will likely increase, as cases of new variants causing reinfection have been increasingly documented [31]. Enhanced genomic and serologic surveillance in congregate settings such as nursing homes, and continued testing of residents ≥90 days post initial diagnosis can provide a clearer understanding of the duration of natural immunity in this population. Finally, we have documented that initial vaccine responses were protective in nursing home settings, but careful determination of the breadth and durability of vaccine versus natural infection responses must be assessed, as such data will also influence testing, clinical risk assessment, and vaccine policies over time, particularly with the rise of new variants of concern [32].

## Declaration of Interests

We declare no competing interests.

## Appendix Supplementary materials

Image, application 1
