# Supplementary material for: Repeat positive SARS-CoV-2 RNA testing in nursing home residents during the initial 9 months of the COVID-19 pandemic: an observational retrospective analysis
Source: Lancet Reg Health Am. 2021 Aug 21;3:100054. doi: 10.1016/j.lana.2021.100054 (PMC8380052; doi:10.1016/j.lana.2021.100054)
Supplement: Supplementary file 1 [file mmc1.zip › Supplemental Figure 2.pdf]

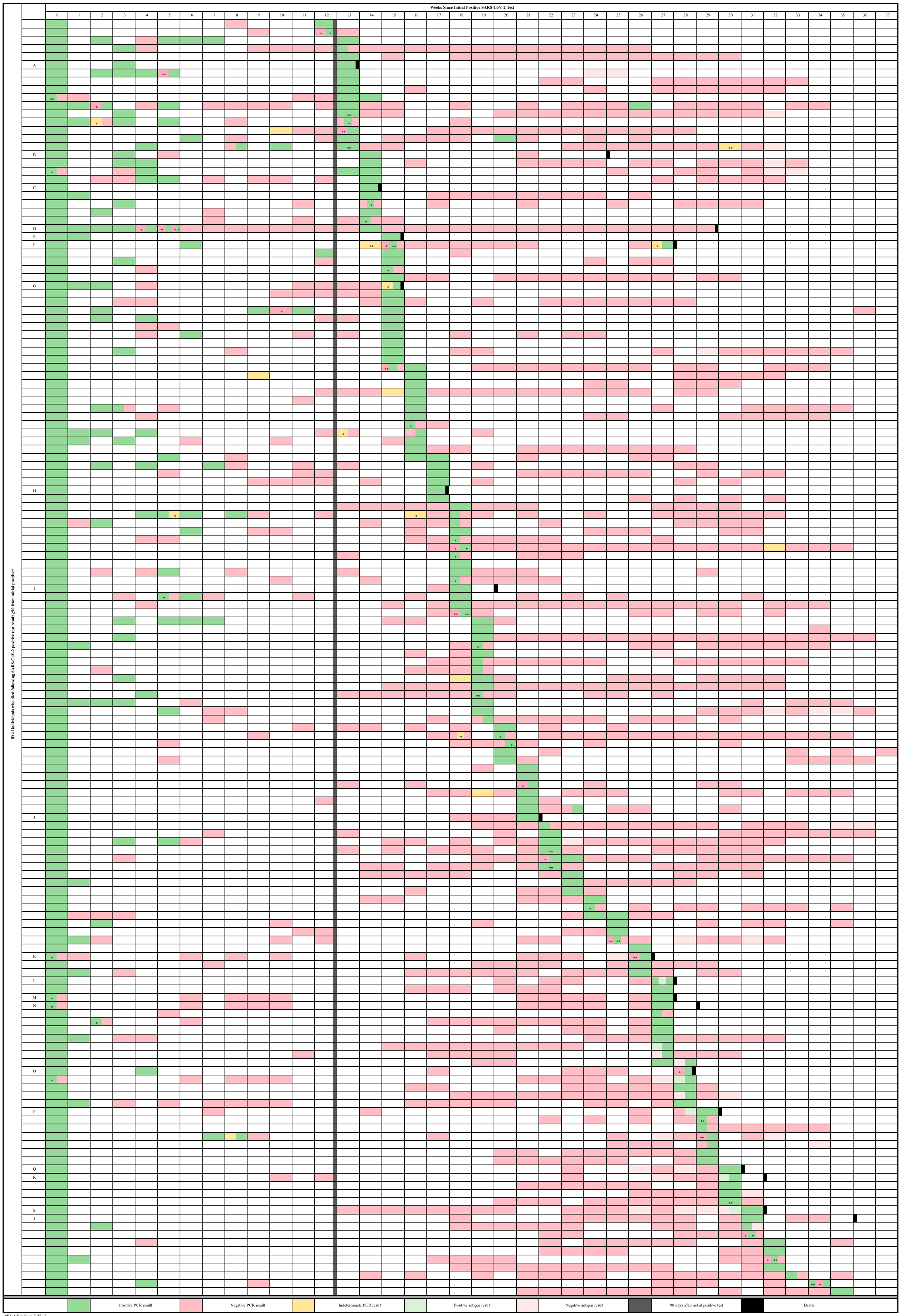

†IDs taken from Table 2.
*This SARS-CoV-2 PCR result was followed by a discordant PCR result within one calendar day.
**This SARS-CoV-2 PCR result was followed by a discordant PCR result within two calendar days.
