# Supplementary material for: Repeat positive SARS-CoV-2 RNA testing in nursing home residents during the initial 9 months of the COVID-19 pandemic: an observational retrospective analysis
Source: Lancet Reg Health Am. 2021 Aug 21;3:100054. doi: 10.1016/j.lana.2021.100054 (PMC8380052; doi:10.1016/j.lana.2021.100054)
Supplement: Supplementary file 1 [file mmc1.zip › mmc1.doc]

**Supplemental Figure 1.** Number of SARS-CoV-2 tests per individual

Histogram of the number of SARS-CoV-2 tests conducted per individual (n=156 residents) over the study period.

**Supplemental Figure 2.** Detailed testing results over 9 months for n=156 residents

Weekly SARS-CoV-2 testing results for nursing home residents with a positive SARS-CoV-2 RT-PCR test ≥90 days following an initial positive SARS-CoV-2 RT-PCR test. Green, red, and yellow represent positive, negative, and intermediate PCR test results respectively. Black hashmarks represent antigen test results. Gray represents the 90-day threshold after the initial positive SARS-CoV-2 RT-PCR test. Black represents the date of reported death. Individuals may have had more than one test during a week. Weeks with discordant results are represented by multiple colors according to the number, results, and sequence of the discordant tests.
